# Bayesian prediction intervals for assessing *P*-value variability in prospective replication studies

**DOI:** 10.1038/s41398-017-0024-3

**Published:** 2017-12-08

**Authors:** Olga Vsevolozhskaya, Gabriel Ruiz, Dmitri Zaykin

**Affiliations:** 1 Biostatistics Department, University of Kentucky, Lexington, KY USA; 2 The Summer Internship Program at the National Institute of Environmental Health Sciences, Research Triangle Park, NC USA; 3 Biostatistics and Computational Biology, National Institute of Environmental Health Sciences, National Institutes of Health, Research Triangle Park, NC USA

## Abstract

Increased availability of data and accessibility of computational tools in recent years have created an unprecedented upsurge of scientific studies driven by statistical analysis. Limitations inherent to statistics impose constraints on the reliability of conclusions drawn from data, so misuse of statistical methods is a growing concern. Hypothesis and significance testing, and the accompanying *P*-values are being scrutinized as representing the most widely applied and abused practices. One line of critique is that *P*-values are inherently unfit to fulfill their ostensible role as measures of credibility for scientific hypotheses. It has also been suggested that while *P*-values may have their role as summary measures of effect, researchers underappreciate the degree of randomness in the *P*-value. High variability of *P*-values would suggest that having obtained a small *P*-value in one study, one is, ne vertheless, still likely to obtain a much larger *P*-value in a similarly powered replication study. Thus, “replicability of *P*-value” is in itself questionable. To characterize *P*-value variability, one can use prediction intervals whose endpoints reflect the likely spread of *P*-values that could have been obtained by a replication study. Unfortunately, the intervals currently in use, the frequentist *P*-intervals, are based on unrealistic implicit assumptions. Namely, *P*-intervals are constructed with the assumptions that imply substantial chances of encountering large values of effect size in an observational study, which leads to bias. The long-run frequentist probability provided by *P*-intervals is similar in interpretation to that of the classical confidence intervals, but the endpoints of any particular interval lack interpretation as probabilistic bounds for the possible spread of future *P*-values that may have been obtained in replication studies. Along with classical frequentist intervals, there exists a Bayesian viewpoint toward interval construction in which the endpoints of an interval have a meaningful probabilistic interpretation. We propose Bayesian intervals for prediction of *P*-value variability in prospective replication studies. Contingent upon approximate prior knowledge of the effect size distribution, our proposed Bayesian intervals have endpoints that are directly interpretable as probabilistic bounds for replication *P*-values, and they are resistant to selection bias. We showcase our approach by its application to *P*-values reported for five psychiatric disorders by the Psychiatric Genomics Consortium group.

## Introduction

Poor replicability has been plaguing observational studies. The “replicability crisis” is largely statistical and while there are limits to what statistics can do, a serious concern is misapplication of statistical methods. Significance testing and *P*-values are often singled out as major culprits, not only because these concepts are easy to misinterpret, but for purported inherent flaws. Variability of *P*-values appears to be underappreciated in the sense that when a small *P*-value is obtained by a given study, researchers commonly suppose that a similarly designed independent replication study is likely to yield a similarly small *P*-value. We will use the term “replication *P*-values”, introduced by Killeen^1^, to mean the *P*-value obtained from subsequent, replicate experiments with the same sample size, taken from the same population. Great variability of replication *P*-values casts doubt on validity of conclusions derived by a study at hand and implies lack of confidence in possible outcomes of any follow-up studies. In reality, one should expect a greater uncertainty in what a replication *P*-value will be. Special prediction intervals for *P*-values, named “*P*-intervals”, have been employed to characterize that uncertainty^2–6^. *P*-intervals have been presented as an objective measure of *P*-value variability, as opposed to subjective judgments reported by researchers in surveys, with the conclusion that the subjective estimates are too narrow and, therefore, researchers tend to underestimate randomness of replication *P*-values^3^. While *P*-intervals have been used mainly as a tool to elucidate flaws of *P*-values, they have also been defended as important additions to *P*-values themselves in publications supportive of *P*-values as universal measures that provide useful summary of statistical tests^6^. It has been suggested that *P*-intervals may serve the purpose of improving *P*-value interpretability, especially in large-scale genomic studies with many tests or in other studies utilizing modern high-throughput technologies^5,6^. For example, in their discussion of *P*-values and their prediction intervals, Lazzeroni and colleagues^6^ argued that the *P*-values “*will continue to have an important role in research*” and that “*no other statistic fills this particular niche*.” They present *P*-intervals not as a way to expose alleged weaknesses of *P*-values but rather as a tool for assessing the real uncertainty inherent in *P*-values.

In our view, the major difficulty with the applications of *P*-intervals for prediction of uncertainty in replication *P*-values is the lack of clear interpretation of their endpoints due to their frequentist construction. Classical prediction intervals, also known as “prediction confidence intervals”, have statistical properties that are similar to the regular confidence intervals (CI’s). Both types of intervals are random and are constructed to cover the replication value (1 − *α*)% of the time, referred to as the coverage property (here, *α* represents the desired type I error rate and (1 − *α*)% represents the desired confidence level). As Lazzeroni and colleagues^5^ noted while discussing results of their simulation experiments, “*By definition, the coverage rate is an average across the distribution*” [of *P*-values]. This statement can be expanded as follows: given a large number of original studies with different *P*-values, if (1 − *α*)% *P*-intervals were to be constructed in each of these original studies regardless of statistical significance of the obtained *P*-value, then the average number of replication *P*-values covered by respective *P*-intervals is expected to be (1 − *α*)%. In this model, there are multiple original studies with a single replication *P*-value for each prediction interval, and it either falls into the interval or it does not. The average is taken over the proportion of times the replication *P*-value falls into the prediction interval. Caveating Lazzeroni et al.’s^5^ discussion, the endpoints of an interval constructed around a *P*-value obtained in any particular study cannot be interpreted in a probabilistic way with regard to a replication *P*-value, because it is either captured by the interval or not and the endpoints of the interval do not represent the range of possible values.

Another difficulty with *P*-interval interpretation arises when it is constructed for a specific *P*-value. The coverage property of *P*-intervals as a long-run average is well-defined for random *P*-values, and the resulting intervals are themselves random. On the other hand, a *P*-interval constructed for a given *P*-value, ${\cal P}$, has specific, fixed endpoints. One way to interpret the endpoints of a particular interval and to relate them to the long-run average definition is to restrict the range of random *P*-values (0 to 1) to a narrow interval around ${\cal P}$, i.e., ${\cal P} \pm \varepsilon$, for some small *ε*. We can think of a process that generates these *P*-values as being the same as in the unrestricted case, but then we would discard any *P*-value outside the ${\cal P}$±*ε* interval and evaluate coverage only for the intervals around *P*-values that are similar to ${\cal P}$. In general, such selection can lead to bias in coverage of the classical interval. For example, Lazzeroni and colleagues reported that the coverage for *P*-values restricted to a specific range could be much smaller than the nominal (1 − *α*)% level expected across all possible values of the *P*-value^5^. Thus, the endpoints of any particular *P*-interval constructed around an obtained *P*-value are not readily interpreted in terms related to the *P*-value at hand or any future values in replication studies.

It is illustrative to follow the reasoning of Neyman, who developed the theory of CI's^7,8^. Neyman starts by approaching the interval estimation from a Bayesian perspective, and describes a posterior distribution of the parameter *θ*, given the data *x* (we will use a different notation, e.g., *μ* in place of *θ*, for consistency with our notation). Neyman writes that this distribution, Pr(*μ*|*x*), “*permits the calculation of the most probable values of the μ and also of the probability that μ will fall in any given interval*,”^7^ for example, *μ*_*L*_ ≤ *μ* ≤ *μ*_*U*_. Neyman notes that the calculation of such a posterior interval requires placing a prior probability distribution on *μ*, something he seeks to avoid through the development of CIs. In the Bayesian set-up, the endpoints *μ*_*L*_ and *μ*_*U*_ are fixed numbers, while *μ* is random. To derive the CI endpoints as functions of random data, *L*(*x*) ≤ *μ* ≤ *U*(*x*), Neyman instead proceeds by working with the probability of the data *x* given the parameter value *μ*: *Pr*(*x*|*μ*). In contrast with posterior intervals, the value *μ* is unknown but constant and the interval endpoints *L*(*x*) and *U*(*x*) are random.

Neyman describes unequivocally the operational usage of CI's as “behavioral”: when a scientist consistently adheres to the rule of deciding to accept that *μ* is contained in every interval computed based on the data *x*, the scientist will be correct (1 − *α*)% of the time in the long-run. He writes that the use of the intervals in practice would consist of collecting data *x*, calculating the endpoints, and “ *stating* that the true value of *μ* lies between [the interval endpoints]”. He stresses that the word ‘‘stating’’ “*is put in italics to emphasize that it is not suggested that we can ‘‘conclude’’ that we can ‘‘conclude’’ that [the true value of μ* = *μ* ^*^ *] is L*(*x*) ≤ *μ* ^*^ ≤ *U*(*x*) *nor that we should ‘‘'believe’' '* *that μ* ^*^ *is actually between L*(*x*) *and U*(*x*)” and continues: “*the probability statements refer to the problems of estimation with which the statistician will be concerned in the future*”, but “*once the sample is drawn and the values of L*(*x*)*, U*(*x*) *determined, the calculus of probability adopted here is helpless to provide answer to the question of what is the true value of μ*”.

Neyman’s description excludes any probabilistic meaning attached to the endpoints of a particular interval: “*after observing the values of the x’s [*…*] we may decide to behave as if we actually knew that the true value [of the parameter μ] were between [L*(*x*) *and U*(*x*)*]. This is done as a result of our decision and has nothing to do with ‘‘reasoning’' or ‘‘conclusion'’ […] The above process is also devoid of any ‘'belief’' concerning the [true value of μ]*”'.

An important point in the preceding discussion of confidence and prediction intervals is that their coverage property is defined as a long-run average of zeros and ones, where “1” indicates an event that a random interval covers the quantity of interest, i.e., a replication *P*-value in the case of *P*-intervals, and “0” indicates that the replication *P*-value is outside that interval. Properly constructed intervals applied repeatedly to independent data sets will result in 1’s occurring with (1 − *α*) frequency. Although it is desirable to have the shortest possible intervals with this property, there is generally no information provided by the interval endpoints about a possible spread of replication *P*-values. However, interpretation of the classical interval endpoints in a meaningful way is warranted from a Bayesian viewpoint. A Bayesian derivation of a classical interval may reveal the tacitly assumed data generating mechanism. We will refer to that mechanism conventionally as an implicit prior distribution. It allows one to interpret the endpoints of a replication *P*-interval as (1 − *α*) probability of capturing the replication *P*-value. The endpoints of a *P*-interval are typically interpreted in a probabilistic fashion without specifying implicit prior assumptions. The following quote from Cumming^2^ gets to the heart of the matter succinctly: “*This article shows that, if an initial experiment results in two-tailed P = 0.05, there is an 80% chance the one-tailed P-value from a replication will fall in the interval (0.00008, 0.44)* […] *Remarkably, the interval*—*termed a P interval*—*is this wide however large the sample size*.” An equivalent statement appears in a *Nature Methods* letter by Halsey and colleagues: “*regardless of the statistical power of an experiment, if a single replicate returns a P-value of 0.05, there is an 80% chance that a repeat experiment would return a P-value between 0 and 0.44*.”^4^ Both statements make use of a specific value, ${\cal P}$ = 0.05, for which the interval is constructed and the endpoints of that interval are described explicitly as probability bounds for possible values of replication *P*-values.

**Fig. 1:**
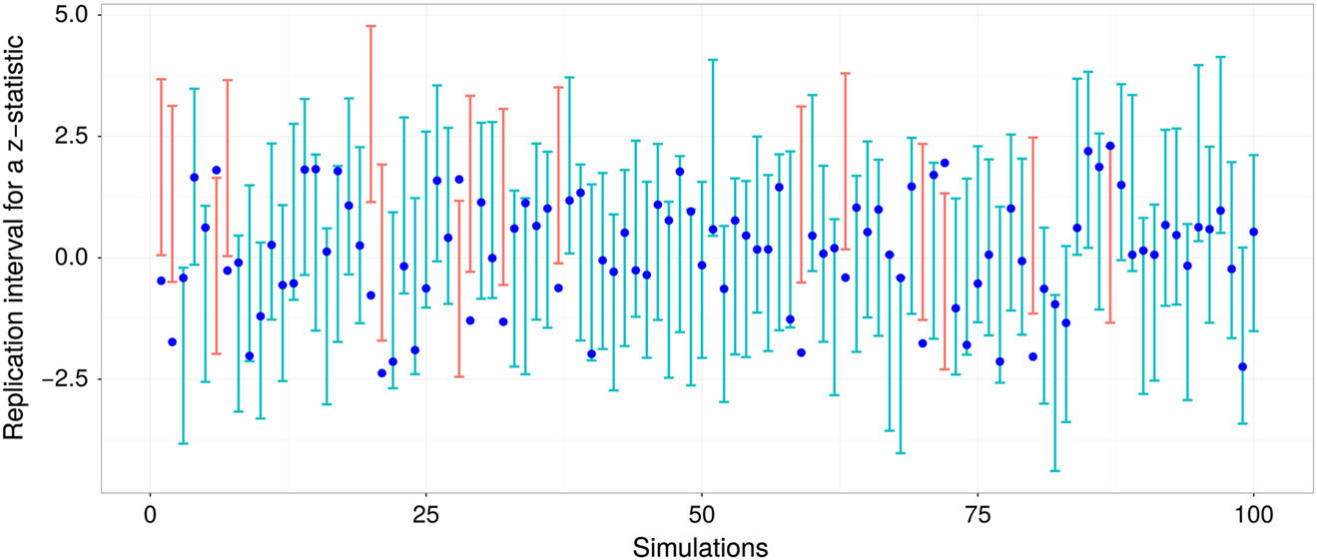
Randomly simulated *Z*-statistics (dots) with the corresponding 80% prediction intervals (vertical error bars). Tests were performed based on two samples (*n*_1_ = *n*_2_ = 50) from two different populations. The difference between population means was a random draw from the standard normal distribution. Pink color highlights intervals that did not capture the value of the future test statistic

To further illustrate possible issues with *P*-interval coverage due to restrictions on the *P*-value range, consider the following example. Suppose one performs a test for the mean difference between two populations and predicts variability of *P*-values in a replication study by constructing the corresponding 80% *P*-interval. If multiple samples are drawn from these populations and 80% *P*-intervals are constructed each time *regardless of whether the observed P-value was significant or not*, the results of these multiple experiments can be summarized graphically by Fig. 1. Each dot in Fig. 1 represents a value of the test statistic from a replication study and error bars show prediction intervals based on the observed *P*-value in the original study. The underlying effect size and sample sizes across studies are kept constant so replication values of the test statistic ranges from about negative two to positive two across all simulated experiments. Pink color highlights *P*-intervals that failed to capture the future value of statistic. Given these results, one can calculate an empirical binomial probability, i.e., the proportion of times a parameter was captured by the interval, which should be close to the stated nominal level. For instance, in Fig. 1, the binomial probability is 84% (16 out of 100 intervals did not capture the future value)—very close to 80% nominal level, given a small number of repetitions.

Now, consider a bit different scenario, in which *P*-intervals are constructed only if the experiment returns a *P*-value close to 0.05, *P*_obt_ ≈ 0.05. That is, all experiments with *P*-values that did not reach statistical significance are discarded and a particular *P*-interval is constructed only if the obtained *P*-value is close to 0.05. Would about 80% of the *P*-intervals constructed around the respective *P*_obt_ still capture the future value of statistic? An intuitive way to think about this scenario is in connection to the publication bias phenomenon, where the actual relationships tend to be weaker in reality than what was claimed in publications, and we may suspect that *P*-intervals should be similarly biased when constructed around non-random, selected subsets of *P*-values.

Further, once a *P*-interval is constructed for a particular *P*_obt_, how can one interpret its bounds? If it is constructed based on an 80% classical prediction interval for a normal test statistic as originally suggested by Cumming^2^, with no regard to prior distribution assumptions, by definition it guarantees that 80% of *P*-intervals will capture a replication *P*-value. That is, the lower and the upper bounds of a *P*-interval do not provide bounds on the range of possible values of a replication *P*-value.

Our main goal in this work is to derive prediction intervals for *P*-values based on the Mixture Bayes approach whose endpoints have a clear probability interpretation for any specific interval constructed based on a given data set. Unlike the classical coverage property, Bayesian intervals based on a posterior *P*-value distribution have the interval endpoints that are directly interpreted as defining a target range to contain a replication *P*-value with probability (1 − *α*).

*P*-values can be viewed as random variables, reflecting variability due to random sampling. This notion goes back to Fisher, whose method of aggregating information from several independent *P*-values is based on recognizing the fact that their product is itself a random variable (and twice the negative logarithm of that product has a chi-square distribution)^9^. The distribution of *P*-value and thus its variability are easily characterized analytically for the basic test statistics and depend on a measure of effect size, such as the value of odds ratio (OR) of disease given exposure vs. nonexposure to a pollutant. The nature of *P*-value randomness may be viewed from a number of angles^10-12^, but the randomness of *P*-value reflects randomness of the respective test statistic. When the effect size is zero, *P*-value of a continuous test statistic is uniformly distributed between zero and one, and as the departure from the null hypothesis increases, the shape of the *P*-value distribution becomes increasingly skewed toward zero (suppl. section S1.) Furthermore, effect sizes can also be thought of as arising from a distribution, (e.g., Equation 11 in Kuo et al.^13^) in which case the *P*-value distribution becomes a weighted average, i.e., a marginal distribution over all possible values of the effect with their respective probabilities as weights.

The idea behind the *P*-intervals is that without specifying any prior knowledge on possible values of the effect size, one can take at face value the magnitude of an obtained *P*-value (*P*_obt_). In other words, there is information about the magnitude of the effect size contained in the magnitude of *P*_obt_, and that information alone can be used to make predictions about a *P*-value obtained in a replication study, denoted as *P*_rep_. As we illustrate with quotes from Cumming^2^ and Hasley^4^, practical applications of *P*-intervals are often factually Bayesian, defaulting to some interpretation about a possible spread of replication *P*-values. Our next goal is to explore relationships between *P*-intervals and Bayesian intervals. These relations are important because not explicitly stating a prior distribution amounts to sweeping a potentially unrealistic prior under the rug. In this work, we give explicit expressions for how the influence of the prior on the *P*-interval diminishes as more data is collected. Eventually, as the sample size increases, the *P*-interval endpoints approach those of the Bayesian intervals, but the sample size requirements depend on the variance of the prior distribution. In observational research, and especially in genetic association studies, where majority of tested hypotheses are effectually false, we find that sample sizes need to be very large.

Further, we show that *P*-intervals can be viewed as a special case of the intervals that we develop: they correspond to the assumption that the product of the sample size *N* and the variance of the prior distribution $s_0^2$ on the standardized effect size (*δ*) is a “large” number, in the sense that if we consider a normal random variable whose variance is $N \times s_0^2$, we could think of its distribution as approximately flat, rather than bell-shaped, in the range that a standardized effect size could be taking (the standardized effect size is defined in units of the standard deviation, e.g., *δ* = *μ*/*σ*). Flat priors are sometimes described as “noninformative,” reflecting lack of researcher’s knowledge or preference about a possible effect size. Yet, far from being uninformative, the flat prior places equal weighting on tiny as well as on large deviations from the null hypothesis. For example, correlation (being the standardized covariance) cannot be outside -1 to 1 interval, so simply acknowledging this range in a prior is already an improvement over an unrestricted prior. If part of the replicability problem lies with the preponderance of tiny effects in reality, the *a priori* assumption of a flat distribution for the effect size implicit in *P*-intervals would tend to result in intervals with the left endpoint that is unrealistically close to zero and thus promote false findings.

How large can $N \times s_0^2$ be assumed to be realistically? Genetic epidemiology and other observational studies routinely test hypotheses that can be viewed conceptually as a comparison of two-sample means. Exposure to an environmental factor or genetic effect of a locus on susceptibility to disease are examples where the presence of effect implies a difference in mean values between subjects with and without disease. In these examples, the effect size can be measured by the log of odds ratio, log(OR). Expecting the majority of effect sizes to be small and the direction of effect to be random, log(OR) can be described by a zero-centered, bell-shaped distribution. It can be shown (Methods section) that the value *δ* = (*μ*_1_ − *μ*_2_)/*σ* for a given value of log(OR) cannot exceed $log({\mathrm{OR}})/\left[ {2\sqrt {2 + (1 + {\mathrm{OR}})/\sqrt {{\mathrm{OR}}} } } \right]$. This implies that the distribution of the standardized effect size is bounded and a considerable spread of *δ* values is unrealistic. To re-iterate this point, the effect size, for example, as measured by log(OR) can be quite large, but the standardized effect size (e.g., $\delta = log({\mathrm{OR}})/\sqrt {{\mathrm{Var}}[{\mathrm{OR}}]}$) can be bounded within a small range of possible values. For example, OR = 4 gives the maximum possible value for *δ* to be about 1/3. Even a very large value OR = 10 results in max(*δ*) ≈ 1/2. Such large ORs are rarely encountered in observational studies^14^, suggesting that realistic variance values $s_0^2$ cannot be very large. Further, as detailed in Methods section, the maximum possible value of *δ* for any OR, no matter how large, cannot exceed ≈ 0.663. This bound places further restrictions on realistic and maximum possible values of the prior variance $s_0^2$, because the prior distribution has to vanish at that bound. Genetic epidemiology studies and genome-wide association scans, in particular, routinely involve massive testing. These studies have uncovered many robustly replicating genetic variants that are predictors of susceptibility to complex diseases. It is also apparent that the vast majority of genetic variants carry effect sizes, such as measured by log(OR), that are very close to zero, and there are commonly only a handful of variants with ORs as large as 1.5. This implies tiny values of $s_0^2$. For example, a reported distribution of effect sizes for the bipolar disorder (BP)^15^ and cancers^16^ translates into the values of the order 10^−6^–10^−5^ for $s_0^2$ (Methods section).

In the next sections, we show how small values of $s_0^2$ render *P*-intervals unfit as a prediction interval for a replication value’s (*P*_rep_) variability and provide a generalization based on the Mixture Bayes approach, which is not constrained to the conjugate model only and provides researchers with the flexibility to specify any desired prior effect size distribution. Our results reveal immunity of the Mixture Bayes intervals to multiple-testing phenomena and to selection bias. When an interval is constructed for a particular value of *P*_rep_, its endpoints can be interpreted as a likely range of the *P*_rep_ values. We contrast the performance of the traditional *P*-intervals relative to the Bayesian-based prediction intervals using results from the Psychiatric Genomics Consortium (PGS)^5^ and conclude with a discussion of the implications of our findings.

## Methods

### Prediction intervals

The *P*-interval can be obtained as a classical prediction interval for the normally distributed test statistics, (*Z*-statistics). The classical interval prediction problem is to probabilistically predict possible values of a future random observation, *X*_*n*+1_, based on a sample of *n* values that have been already obtained, *X*_1_,…,*X*_*n*_. In the case of the *Z*-statistic, the information about the effect (e.g., the population mean) is summarized by the sample average, $\bar X$. Although on the surface, prediction of a future “replication” value, *Z*_rep_ is based on a single obtained test statistic, *Z*_obt_, that statistic, as well as its corresponding *P*-value, *P*_obt_, depend on all *n* sample observations. Moreover, in cases such as this, $\bar X$, being a sufficient statistic, contains all information about the unknown mean (i.e., the effect size) available from the data. Therefore, based on a *P*-value as the only summary of data, it is possible, at least for standardized effect sizes, to obtain the full Bayesian posterior distribution and to characterize uncertainty about the effect size values. This conversion of statistics or *P*-values to posterior distributions requires one to augment information contained in *P*-values with a prior distribution on the standardized effect size. This approach is quite general, because while effect size may be measured by different types of statistics, such as the difference of two-sample means, or the logarithm of the OR, the summary of the effect present in the data is captured by the same *Z*-statistic, and it is the type of the test statistic that determines the interval properties, rather than a particular measure of the effect size.

The prediction distribution for the statistic *Z*_rep_ relates to one-sided *P*-value as *P*_rep_ = 1−Φ(*Z*_rep_) and has a normal distribution, Φ(*z*_obt_,2), where Φ(⋅) is the standard normal cumulative distribution function (CDF). Thus, the classical *P*-interval is constructed as:

1 $$z_{{\mathrm{obt}}} \pm z_{(1 - \alpha /2)}\sqrt 2 ,$$

where *z*_(1−*α*/2)_ is the 1−*α*/2 quantile of the standard normal distribution. This distribution does not depend on the actual mean of $Z$, which is $\sqrt {N} \times \delta$. The reason for that becomes apparent when the *P*-interval is derived as a Bayesian prediction interval. For a normally distributed *Z*-statistic, $Z\sim N(\mu ,1)$, assume the conjugate model, that is, $\mu \sim \sqrt {N} \times \Phi (m_0,s_0^2)$. Then, the posterior distribution for the mean of *Z*_obt_ is normal Φ(*θ*, *s* ^2^), where

2 $$\theta \left| {Z_{{\mathrm{obt}}}} \right. = \frac{{\frac{{m_0}}{{\sqrt {N} s_0^2}} + Z_{{\mathrm{obt}}}}}{{s^2}}$$

3 $$s^2\left| {Z_{{\mathrm{obt}}}} \right. = \left[ {\frac{1}{{Ns_0^2}} + 1} \right]^{ - 1},$$

and the prediction distribution for *Z*_rep_ is Φ(*θ*,1 + *s* ^2^). Therefore, the *P*-interval based on the distribution Φ(*z*_obt_,2) is a Bayesian interval that implicitly assumes that $N \times s_0^2 \to \infty$, which makes *s* ^2^|*Z*_obt_ = 1 and the prediction distribution for *Z*_rep_ equal to Φ(*θ*,2). We refer to the resulting intervals as the Conjugate Bayes intervals. The endpoints of these intervals are given by

4 $$\theta \pm z_{(1 - \alpha /2)}\sqrt {s^2 + 1}$$

5 $$\equiv Z_{{\mathrm{obt}}}\frac{{\sigma _0^2}}{{1 + \sigma _0^2}} + \frac{{m_0}}{{1 + \sigma _0^2}} \pm z_{(1 - \alpha /2)}\sqrt {1 + \frac{{\sigma _0^2}}{{1 + \sigma _0^2}}} ,$$

$$\sigma _0^2 = Ns_0^2$$

Derived as a Bayesian prediction interval through the conjugate model, the endpoints of a *P*-interval in Eq. (5) can now be interpreted as bounds of the supposed likely range of replication *P*-values within a given probability (e.g., 80%). However, the conjugate model is restrictive in that a specific prior distribution has to be assumed, which may not provide an adequate representation of external knowledge about the effect size distribution. It also limits construction of the intervals to *P*-values derived from statistics for which there are known conjugate priors. Here, we introduce a more flexible approach, the Mixture Bayes, without these restrictions. The Mixture Bayes intervals can be constructed for *P*-values derived from statistics whose distribution is governed by a parameter *γ* that captures deviation from the usual point null hypothesis, *H*_0_, and has the form $\sqrt {N} \times \delta$ or its square, *N *× *δ* ^2^. This includes normal, chi-squared, Student’s *t* and F-statistics. We partition the prior distribution of $\gamma$ into a finite mixture of values *δ*_1_, *δ*_2_,…, *δ*_*B*_ with the corresponding prior probabilities, *Pr*(*δ*_*i*_). As an example, let *P*-value be derived from an F-test for comparison of two-sample means, with the corresponding sample sizes *n*_1_ and *n*_2_. Let *N *= 1/(1/*n*_1_ + 1/*n*_2_). For *i*-th prior value of effect, a statistic based on sampling values of $T = (\bar X_1 - \bar X_2)^2/\hat \sigma ^2$has a noncentral F-distribution, with the noncentrality

6 $$\gamma _i = N\left[ {(\mu _1 - \mu _2)/\sigma } \right]_i^2 = N\delta _i^2,$$

and the degrees of freedom df_1 _= 1, df_2_ = *n*_1_ + *n*_2 _− 2:

7 $$T\sim f\left( {T = t\left| {\gamma _i,{\mathrm{df}}_1,{\mathrm{df}}_2} \right.} \right),$$

where *f* is the density of the noncentral F-distribution. The posterior distribution is a mixture,

8 $$Pr\left( {\delta _j^2\left| T \right. = t} \right) = \frac{{Pr(\delta _j^2)f\left( {T = t\left| {\gamma _j} \right.,{\mathrm{df}}_1,{\mathrm{df}}_2} \right)}}{{\mathop {\sum}\nolimits_{i = 1}^B {Pr(\delta _i^2)f} \left( {T = t\left| {\gamma _i} \right.,{\mathrm{df}}_1,{\mathrm{df}}_2} \right)}},$$

with the posterior mean

9 $$\theta = \mathop {\sum}\limits_{i = 1}^B \delta _i^2\,Pr\left( {\left. {\delta _i^2} \right|{\mathrm{P}} - {\mathrm{value}}} \right).$$

Next, we obtain the CDF of the prediction distribution for the replication statistic, *T*_rep_, as

10 $$\begin{array}{ccccc}\\ F_p(x) = & \mathop {\sum}\limits_j^B Pr\left( {\delta _j^2\left| {T_{{\mathrm{obt}}}} \right.} \right){\int}_0^x f\left( {T_{{\mathrm{rep}}}\left| {\gamma _j} \right.,{\mathrm{df}}_1,{\mathrm{df}}_2} \right)dT_{{\mathrm{rep}}}\\ =& \mathop {\sum}\limits_j^B Pr\left( {\delta _j^2\left| {T_{{\mathrm{obt}}}} \right.} \right)F\left( {T_{{\mathrm{rep}}} = x\left| {\gamma _j} \right.,{\mathrm{df}}_1,{\mathrm{df}}_2} \right).\end{array}$$

Then, the Mixture Bayes interval endpoints are derived from the quantiles of this CDF that are given by $F_p^{ - 1}(x)$.

We have developed a user-friendly software tool for implementation of our Mixture Bayes approach, available at https://github.com/dmitri-zaykin/bayesian-PValue-Prediction-Intervals. The software allows users to construct a Mixture Bayes prediction interval for a *P*-value from the standard normal, Student’s *t*, chi-square or an F-statistic. For a *P*-value based on the standard normal or a *t* distribution, users have a choice between the conjugate normal model and the tabulated prior effect size distribution. For the F and the chi-squared test, no conjugate model exists, but prior values can be specified in a tabulated manner.

### Prior variance for the standardized logarithm of the OR

Genetic epidemiology and other observational studies routinely test hypotheses conceptually related to a comparison of two-sample means. Effect size is often measured by the log of OR, which can be related to the difference in means (that become frequencies, *p*_1_ and *p*_2_, in the case of binary variables) as $p_1 - p_2 \approx log({\mathrm{OR}}){\kern 1pt} \tilde p(1 - \tilde p)$, where $\tilde p$ is the pooled frequency. Distribution of *P*-values for commonly used test statistics depends on the product of the sample size, (*N* or $\sqrt {N}$), and a measure of effect size, *μ*, scaled by the variance *σ* ^2^ (or *σ*), i.e. *δ* = *μ*/*σ*. For example, when the outcome is a case/control classification and the predictor is also binary, the standardized effect size can be expressed in terms of the correlation (*R*) times the sample size as follows:

11 $$\gamma = \sqrt {N} \times \frac{\mu }{\sigma } = \sqrt {N} \times \delta = \sqrt {N} \times R$$

12 $$= \sqrt {N} \times \frac{{p_1 - p_2}}{{\sqrt {\tilde p(1 - \tilde p)\left[ {v(1 - v)} \right]^{ - 1}} }},$$

where *v* is the proportion of cases in the sample. In terms of the logarithm of the odds ratio, OR,

13 $$\gamma = \sqrt {N} \times \delta = \sqrt {N} \times \frac{{log({\mathrm{OR}})}}{{\sqrt {\frac{1}{v}\frac{1}{{p_1(1 - p_1)}} + \frac{1}{{1 - v}}\frac{1}{{p_2(1 - p_2)}}} }}$$

14 $$\approx \sqrt {N} \times \frac{{log({\mathrm{OR}})}}{{\sqrt {\left[ {\tilde p(1 - \tilde p)v(1 - v)} \right]^{ - 1}} }}.$$

For a given value of OR, the standardized effect size *δ* cannot exceed the value *δ*_max_(OR) that we obtained by maximizing the right hand side of Eq. (13) as:

15 $$\delta _{{\mathrm{max}}}({\mathrm{OR}}) = \frac{{ln({\mathrm{OR}})}}{{2\sqrt {2 + \frac{{1 + {\mathrm{OR}}}}{{\sqrt {{\mathrm{OR}}} }}} }}.$$

Let $F^{ - 1}\left( { \cdot \left| {\mu _0} \right.,s_0^2} \right)$ denote the inverse CDF of the conjugate prior distribution. Writing *Pr*(OR ≥ *x*) = *β* and assuming a symmetric distribution of the effect size around zero, i.e., *m*_0 _= 0, we can relate the value *δ*_max_ to the prior variance of the conjugate model in the following way:

$$\delta _{{\mathrm{max}}}({\mathrm{OR}}) = \sqrt {s_0^2} {\kern 1pt} F^{ - 1}\left( {1 - \beta \left| {0,1} \right.} \right).$$

The maximum spread for the conjugate prior distribution is, therefore, obtained when its variance is equal to

16 $$s_0^2 = \left[ {\frac{{\delta _{{\mathrm{max}}}({\mathrm{OR}})}}{{F^{ - 1}(1 - \beta \left| {0,1} \right.)}}} \right]^2.$$

It should be noted that Eq. (15) gives the maximum *δ* value for a given value of OR, however, it is not monotone in OR. The maximum possible value of *δ*_max_(OR) can be found to be at OR ≈ 121.35. Curiously, this value of OR implies *δ*_max_(OR) value equal to the Laplace Limit constant, 0.662743…

In Eq. (3), we showed that a classical *P*-interval is equivalent to a Bayesian prediction interval if $N \times s_0^2 \to \infty$. Given a bounded nature of the standardized effect size distribution, how large can prior variance $N \times s_0^2$ be expected in reality? Park et al.^16^ reported distribution of effect sizes for breast, prostate and colorectal (BPC) cancers in terms of a table, giving the numbers of different loci (*L*_*i*_) with the corresponding values of OR_*i*_. Using the same approach, Chen et al.^15^ provided the effect size distribution for the BP risk loci. Assuming the total number of independent variants to be *M* = 300,000, proportions of associated loci are *w*_*i*_ = *L*_*i*_/*M*. We assumed the average OR among non-associated loci to be 1.005 (or its inverse for the negative part of the log(OR) distribution). The variance $s_0^2$ was calculated as $\mathop {\sum}\nolimits_i w_i(\gamma _i/\sqrt {2N} - m_w)^2$, where $m_w = \mathop {\sum}\nolimits_i w_i\gamma _i/\sqrt {2N}$, and gave the value ≈ 5 × 10^−6^ for both cancer and the BP disorder risk loci. Thus, *N* needs to be about 50,000 for $s_0^2 \times N$ to reach 1/2.

In the next section, we explore *P*-interval and Mixture Bayes interval performance for the different values of prior variance $\sigma _0^2 = s_0^2 \times N$ and under various forms of selection. In experiments with multiple statistical tests, it is a common practice to select most promising results: tests that yielded the smallest *P*-values would be commonly selected. One may also select results with the largest effect sizes as tentatively most promising for a follow-up. This selection induces a “selection bias”, for example, the largest estimated effect size in the original study would tend to be smaller once re-evaluated in a replication experiment, a phenomenon also known as the winner’s curse^17^. This would reflect the fact that the actual population effect size would tend to be over-estimated due to selection of the best outcome from the original study. An intuitive way of seeing why a selection bias would be present is to imagine a multiple-testing experiment where none of the tested predictors have any relation to the outcome. When one selects a predictor that showed the maximum estimated effect size, there will obviously be a bias, because the true effect size is zero. But this type of bias would also be present if the underlying effect sizes are non-zero for some or all of the predictors. Selection bias is difficult to correct for in the frequentist setting, but Bayesian analysis can be robust to this bias^18^. It is expected that the performance of frequentist-based *P*-intervals may suffer under selection while Bayesian-based intervals may not be affected by it. Thus, we investigated several types of selection and the resulting potential bias, measured by the proportion of times an interval captures *P*_rep_ relative to the stated nominal level (e.g., 80%).

## Results

Table 1 summarizes empirical binomial probabilities of the 80% prediction intervals for a standardized effect size $\sqrt {N} \times \delta$ under different types of *P*-value selection (simulation study set-up is detailed in Supplementary Information). The observed *P*-value was based on a two-sample *Z*-test and was thresholded according to the following selection rules: (i) no selection, i.e., a prediction interval is constructed for a randomly observed *P*-value; (ii) selection of *P*-values around a value, e.g., ${\cal P} \approx 0.05$, i.e., prediction intervals are constructed only for *P*-values that were close to the 5% significance level; (iii) selection of *P*-values that are smaller than a threshold, e.g., ${\cal P} < 0.05$. Empirical binomial probabilities were calculated based on 50,000 simulations, using three different methods: (a) a conjugate Bayesian model assuming normal prior distribution for the observed value of a test statistic, $Z_{{\mathrm{obt}}}\sim \Phi (0,\sigma _0^2)$, where $\sigma _0^2 = s_0^2 \times N$; (b) our Mixture Bayes approach with the same prior as for the conjugate model; and (c) the original *P*-interval proposed by Cumming^2^.

**Table 1:** Binomial probabilities for 80% prediction intervals, using a two-sample *Z*-test

Type of *P*-value selection	Prior variance, $\left( {\sigma _0^2} \right)$	Conjugate Bayes	Mixture Bayes	*P*-interval
0 ≤ *P*-value ≤ 1	0.25	80.1%	80.2%	80.2%
(no selection)	0.50	80.0%	80.0%	79.9%
	1.00	80.0%	80.0%	80.0%
	3.00	80.4%	80.4%	80.4%
	5.00	80.2%	80.2%	80.3%
	10.00	80.1%	80.1%	80.1%
0.045 ≤ *P*-value ≤ 0.055	0.25	79.8%	79.8%	58.4%
	0.50	80.1%	80.1%	66.7%
	1.00	79.8%	79.8%	73.5%
	3.00	80.0%	80.0%	80.2%
	5.00	79.9%	79.9%	80.7%
	10.00	80.1%	80.1%	80.8%
0 ≤ *P*-value ≤ 0.05	0.25	80.0%	80.0%	46.0%
	0.50	80.1%	80.1%	55.4%
	1.00	80.2%	80.2%	65.5%
	3.00	79.8%	79.8%	75.7%
	5.00	80.4%	80.4%	78.4%
	10.00	80.3%	80.3%	79.5%
0 ≤ *P*-value ≤ 0.001	0.25	80.1%	80.1%	17.0%
	0.50	80.1%	80.1%	29.7%
	1.00	80.0%	79.9%	47.6%
	3.00	80.0%	80.0%	70.2%
	5.00	79.9%	79.9%	75.4%
	10.00	79.7%	79.8%	78.2%
5 × 10 ^− 8^ ≤ *P*-value ≤ 5 × 10 ^− 7^	3.00	80.1%	80.1%	62.8%
	5.00	79.5%	79.5%	72.6%
	10.00	79.8%	79.8%	78.3%
5 × 10 ^− 9^ ≤ *P*-value ≤ 5 × 10 ^− 8^	3.00	80.0%	80.0%	60.6%
	5.00	79.9%	80.0%	71.8%
	10.00	80.2%	80.2%	78.1%

The table illustrates the effect of thresholding, applied to observed *P*-values, e.g., selection of statistically significant *P*-values at 5% level, on binomial probabilities

Mixture Bayes intervals were included in these simulations to check how well they approximate a continuous prior distribution assumed by the conjugate intervals. We used mixture components with the length *σ*_0_/8 for every component and truncated the normal prior at 10^−6^ and 1−10^−6^ quantiles. This provided us with sufficient accuracy and resulted in the number of mixture components, *B*, equal to 76 for all values of $\sigma _0^2$.

Table 1 clearly indicates that all three construction methods have the correct coverage (~80%) if a prediction interval is calculated for a randomly observed *P*-value ∈[0,1]. However, selection and small prior variance both impair performance of *P*-intervals. For instance, if an interval is constructed for a *P*-value < 0.001 and $\sigma _0^2 = 0.25$, the coverage of the traditional non-Bayesian *P*-interval may be as low as 17%. This poor coverage is due to a combination of both the selection bias and the implicit assumption that prior variance of $\sigma _0^2$ ranges from negative infinity to positive infinity, which leads to the left-side *P*-interval endpoint being too close to zero. However, even for large values of prior $\sigma _0^2$, the *P*-interval has poor coverage when constructed for *P*-values around genome-wide significance levels (e.g., *P*-value <1.5 × 10^−7^). On the other hand, for large *P*-values the coverage of *P*-intervals becomes greater than the nominal (1 − *α*)% value. This is a consequence of the fact that *P*-interval’s width depends on the magnitude of *P*-values and as *P*-values become larger, the width of the interval increases as well. For example, given the prior variance $N \times s_0^2 = 0.5$, the width of *P*-intervals and the Bayesian intervals coincides at *P*-value = 0.446 (hence, the Bayesian intervals are wider than *P*-intervals at values smaller than 0.446). The *P*-interval around $P$ = 0.446 is: 0.147 ≤*P *≤ 0.995, while the Bayesian interval is 0.110 ≤ *P* ≤ 0.958.

**Fig. 2:**
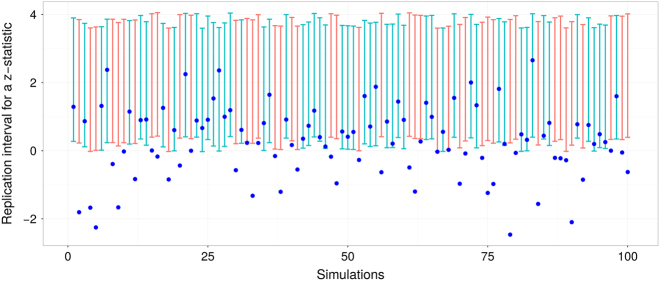
Selection bias influences the performance of prediction intervals. Eighty-percent prediction intervals constructed for ${\mathrm{P}}_{{\mathrm{obt}}}\sim 0.05$ have noticeably poorer performance relative to the ones constructed for a random statistic

To illustrate implications of decrease in *P*-interval coverage for *P*-values less than 0.05, we replicated Fig. 1 under the assumption that a *P*-interval is constructed only for *P*-values close to 0.05, ${\cal P} \approx 0.05$. The results are summarized in Fig. 2 and show how *P*-intervals are becoming increasingly likely to miss *P*_rep_ values altogether. The underlying effect size was kept the same in both figures and blue dots that represent values of *z*_rep_ have similar range. Restricting *P*-values to be close to 0.05 induces selection bias, causing overestimation of the underlying effect size (that is, *z*_obt_ will tend to be larger than it should be, given the effect size magnitude) and a vertical shift in *P*-intervals. Bias in coverage can be potentially removed by extending the interval endpoints by a correct amount, but the appropriate size of the interval appears difficult to determine analytically in a general way.

Similar conclusions regarding coverage can be drawn if a prediction interval is constructed for the most significant *P*-value out of *L* tests (Table 2). That is, if a *P*-interval is constructed for the smallest *P*-value out of *L *= 10, 100, or 1000 tests, both Bayesian methods have the correct coverage and are immune to the selection bias. The non-Bayesian *P*-interval approach, however, once again performs poorly if the prior variance is small. Additionally, as the number of tests increases, out of which a minimum *P*-value is selected, the *P*-interval coverage is becoming increasingly off the 80% mark.

**Table 2:** Binomial probabilities for 80% prediction intervals, using a two-sample *Z*-test

Number of tests	Prior variance $\left( {\sigma _0^2} \right)$	Conjugate Bayes	Mixture Bayes	*P*-interval
*L* = 10	0.25	80.4%	80.4%	63.8%
	0.50	79.9%	79.9%	66.2%
	1.00	80.6%	80.6%	70.4%
	3.00	80.0%	80.0%	75.1%
	5.00	80.1%	80.1%	76.7%
	10.00	80.1%	80.1%	78.3%
*L* = 100	0.25	79.8%	79.8%	35.7%
	0.50	80.2%	80.2%	42.3%
	1.00	79.9%	79.9%	51.1%
	3.00	79.6%	79.6%	65.1%
	5.00	80.0%	80.0%	70.0%
	10.00	79.8%	79.8%	74.5%
*L* = 1000	0.25	80.0%	80.1%	16.9%
	0.50	79.9%	79.8%	23.9%
	1.00	80.0%	79.9%	35.0%
	3.00	80.0%	79.9%	55.5%
	5.00	79.7%	79.6%	63.1%
	10.00	80.2%	80.1%	70.7%
*L* = 10,000	0.25	80.1%	80.1%	07.2%
	0.50	80.1%	80.0%	12.9%
	1.00	79.8%	79.6%	23.1%
	3.00	79.7%	79.1%	46.2%
	5.00	80.2%	79.6%	56.5%
	10.00	80.2%	79.5%	66.5%

The table illustrates the effect of selecting the most significant *P*-value (out of *L* tests) on *P*-interval coverage

*P*-intervals are a special case of our Mixture Bayes intervals, and can be obtained by specifying the prior distribution for $\delta$ as a zero-mean normal with the prior variance $s_0^2$ such that $\sigma _0^2 = N \times s_0^2$ is very large. When *P*-values are selected based on a cutoff value or their magnitude, *P*-intervals can still be a poor approximation to a distribution with $\sigma _0^2$ as large as 10. For example, the last row of Table 2 demonstrates that *P*-intervals are still biased for $\sigma _0^2 = 10$ in terms of the coverage when constructed for the minimum *P*-value taken from multiple-testing experiments with 10,000 tests. Multiple-testing on the scale of genome-wide studies would further degrade the coverage of *P*-intervals. This places specific restrictions on how large $s_0^2$ can be. For the zero-mean normal prior, $s_0$ = 0.66/3 is still unreasonably large, and in general, even for prior distributions concentrated at these bounds, $s_0^2 \le (U - L)^2/4$ by Popoviciu’s inequality.

We next explored the effect of prior variance mis-specification on the coverage of the Bayesian-type prediction interval when it is constructed for the most significant result out of *L* tests. Two scenarios were considered: under-specification ($\sigma _0^2/2$) and over-specification $(2 \sigma _0^2)$ of the prior variance $\sigma _0^2 = Ns_0^2$. The results are summarized in Table 3 and indicate that in terms of the coverage it is safer to over-specify values of the prior variance than to under-specify them. The conjugate model with *m*_0_ = 0 gives the intervals in the following form

17 $$Z_{{\mathrm{obt}}}\frac{{\sigma _0^2}}{{1 + \sigma _0^2}} \pm z_{(1 - \alpha /2)}\sqrt {1 + \frac{{\sigma _0^2}}{{1 + \sigma _0^2}}} ,$$

indicating that $\sigma _0^2$ values that are too small pull the interval mean excessively toward zero while at the same time reducing its proper length.

**Table 3:** The effect of the prior variance mis-specification on the coverage of Bayesian-type prediction intervals

Number of tests	Prior variance	Bayesian	Bayesian	*P*-interval
	$\left( {\sigma _0^2} \right)$	$\left( {\sigma _0^2/2} \right)$	$\left( {2\sigma _0^2} \right)$	
*L* = 1	0.5	77.5%	81.7%	80.3%
	1	76.5%	81.5%	79.7%
*L* = 10	0.25	77.6%	81.1%	63.8%
	0.5	75.9%	80.8%	66.2%
*L* = 100	3	70.4%	77.8%	65.1%
	1	72.1%	77.2%	51.1%
*L* = 1000	0.25	76.4%	78.2%	16.9%
	0.5	72.9%	75.8%	23.9%
*L* = 10,000	3	62.0%	72.9%	46.2%
	10	69.5%	77.1%	66.5%

Unlike the regular Bayesian model, our Mixture Bayes approach is not limited to conjugate priors and prediction intervals can be constructed for any *P*-value stemming from statistics other than the normal *Z*-test. Additionally, the Mixture Bayes approach allows the use of any prior distribution and enjoys the same coverage properties as the conjugate-Bayes prediction intervals, that is, resistance to multiple testing and selection bias (Supplementary Tables S1 and S2).

To illustrate the interpretation of the prediction intervals and contrast the performance of the Bayesian-based intervals to the classical *P*-intervals, we replicated part of Table 1 in Lazzeroni et al.^5^, who considered recent findings from the Psychiatric Genomics Consortium (PGC) for attention deficit-hyperactivity disorder (ADHD), autism spectrum disorder (ASD), bipolar disorder (BPD), major depressive disorder (MDD), and schizophrenia. The consortium reported four single-nucleotide polymorphisms (SNPs) associated with these psychiatric disorders but, for illustrative purposes, we constructed prediction intervals only for a single SNP, rs2535629. We used four different methods to calculate prediction intervals: (i) the conjugate Bayesian model with the estimated prior variance, $s_0^2$, based on the results from Chen et al.^15^ (see Methods section); (ii) Mixture Bayes approximation to this continuous conjugate normal prior, using the same variance, $s_0^2$; (iii) Mixture Bayes approach with the BP effect size distribution reported in Chen et al. as a prior (without assuming the conjugate model); and (iv) prediction intervals suggested by Lazzeroni et al. (which are equivalent to Cumming’s *P*-intervals for one-sided *P*-values). We note that prediction intervals given in Table 1 of Lazzeroni et al. are constructed for a two-sided hypotheses test on the −$\mathop {{log}}\nolimits_{10}$(*P*-value) scale. However, follow-up studies target replication of the directional effects. For example, if a study reports a risk allele for a phenotype of interest and a replicatioin study finds the effect to be protective, one can not conclude that the follow-up study replicated the original report. Thus, one-sided tests would be more appropriate in follow-up studies and prediction intervals for one-sided *P*-values should be of interest. To transform prediction intervals for a two-sided *P*-value in Table 1 of Lazzeroni et al. into prediction intervals for a one-sided *P*-value, one needs to subtract logarithm based ten of two ($\mathop {{log}}\nolimits_{10} (2)$) from both prediction interval bounds. Further, to highlight differences in the performance of the Bayesian-based intervals and *P*-intervals, we transformed prediction bounds in Lazzeroni et al. from -$\mathop {{log}}\nolimits_{10}$(*P*-value) scale to *P*-value scale by raising ten to the negative logarithm based ten of the bounds power.

Table 4 summarizes the results. For all psychiatric disorders, lower bounds of the 95% prediction intervals for *P*-values based on the approach suggested by Lazzeroni et al.^5^ are smaller than the ones from the Bayesian-based methods. For instance, Lazzeroni and colleagues concluded that in a similarly powered replication of the original PGC design, a *P*-value for an association between rs2535629 and ADHD could be as low as $2.57 \times 10^{ - 5}$, given the observed one-sided *P*-value of 0.1005. Our interval results portray a less optimistic picture with the *P*-value lower bound for ADHD equal to 0.023. Similar observations hold for psychiatric disorders with significant observed *P*-values. For example, in Lazzeroni et al. the BP is concluded to be likely to yield a *P*-value between 1.69 × 10^−13^ and 0.04 and thus could reach genome-wide significance (*P*-value <10^−8^) at a replication study. Mixture Bayes prediction interval based on the reported effect size distribution^15^ suggests a higher lower bound for BP replication *P*-value of 6.92 × 10^−7^, concluding that a second, identical implementation of the original PGC design would be unlikely to yield any *P*-values <10^−8^. This difference in the spread of possible replication *P*-values highlights the implicit prior assumption built into the *P*-intervals that large effect sizes are as likely to be observed as small ones.

**Table 4:** Revised predictions based on recent results from the Psychiatric Genomics Consortium with the prior effect size distribution estimated for the bipolar disorder susceptibility loci

SNP	Disorder	Cases	Controls	One-sided *P*-value	Prediction intervals for *P*_rep_			
					Conjugate Bayes^a^	Mixture Bayes^a^	Mixture Bayes^b^	Lazerroni et al.
rs2535629	ADHD	2787	2635	0.1005	(0.023, 0.977)	(0.023, 0.977)	(0.023, 0.977)	(2.57e-5, 0.93)
	ASD	4949	5314	0.098	(0.022, 0.977)	(0.022, 0.977)	(0.022, 0.977)	(2.39e-5, 0.93)
	BP	6990	4820	3.305e-06	(0.017, 0.977)	(0.017, 0.977)	(6.92e-7, 0.93)	(1.69e-13, 0.04)
	MDD	9227	7383	0.000108	(0.016, 0.977)	(0.016, 0.977)	(5.25e-6, 0.95)	(4.89e-11, 0.18)
	Schizophrenia	9379	7736	3.355e-05	(0.015, 0.977)	(0.015, 0.977)	(3.98e-7, 0.95)	(6.92e-12, 0.11)
	All	33,332	27,888	1.27e-12	(0.001, 0.871)	(0.001, 0.871)	(2.2e-18, 1.5e-5)	(7.41e-23, 1.17e-5)

*ADHD* attention deficit-hyperactivity disorder, *ASD* autism spectrum disorder, *BP* bipolar disorder, *MDD* major depressive disorder

^a^The prior effect size distribution using the conjugate model with the variance estimated based on the tabulated values of effect sizes reported in Chen et al.

^b^The prior effect size distribution specified directly by the estimates reported in Chen et al.

Nonetheless, similar to conclusions in Lazzeroni et al., the association of rs2535629 with BP appears to be a promising signal. Also, similar to the conclusions in Lazzeroni et al., the combined study of all psychiatric disorders is predicted to perform better than replication studies of individual phenotypes (95% Mixture Bayes prediction interval: ($2.2 \times 10^{ - 18},1.5 \times 10^{ - 5}$); 95% *P*-interval: ($7.4 \times 10^{ - 23},1.2 \times 10^{ - 5}$)).

While it is expected that different diseases would have different effect size distributions, we wanted to check the robustness of our results to prior mis-specification and utilized available effect size distribution given in Park et al.^16^ for cancers. This assumes that the effect size distribution in terms of ORs has common main features for different complex diseases, namely, that it is L-shaped with the majority of effect sizes that can be attributed to individual SNPs being very small, and that the frequency of relatively common variants with increasingly large values of OR quickly dropping to zero for OR as large as about 3. The modified intervals are reported in Table 5. While Mixture Bayes intervals become somewhat different from those derived using the effect size distribution for BP, their bounds are much more similar to each other than to the bounds of *P*-intervals.

**Table 5:** Revised predictions based on recent results from the Psychiatric Genomics Consortium with the prior effect size distribution estimated for cancer risk loci

SNP	Disorder	Cases	Controls	One-sided *P*-value	Prediction intervals for $P_{rep}$			
					Conjugate Bayes^a^	Mixture Bayes^a^	Mixture Bayes^b^	Lazerroni et al.
rs2535629	ADHD	2787	2635	0.1005	(0.023, 0.977)	(0.023, 0.977)	(0.023, 0.977)	(2.57e-5, 0.93)
	ASD	4949	5314	0.098	(0.022, 0.977)	(0.022, 0.977)	(0.022, 0.977)	(2.39e-5, 0.93)
	BP	6990	4820	3.305e-06	(0.017, 0.977)	(0.017, 0.977)	(5.89e-9, 0.93)	(1.69e-13, 0.04)
	MDD	9227	7383	0.000108	(0.016, 0.977)	(0.016, 0.977)	(7.41e-5, 0.98)	(4.89e-11, 0.18)
	Schizophrenia	9379	7736	3.355e-05	(0.015, 0.977)	(0.015, 0.977)	(9.33e-7, 0.95)	(6.92e-12, 0.11)
	All	33,332	27,888	1.27e-12	(0.001, 0.871)	(0.001, 0.871)	(8.91e-20, 4.2e-5)	(7.41e-23, 1.17e-5)

*ADHD* attention deficit-hyperactivity disorder, *ASD* autism spectrum disorder, *BP* bipolar disorder, *MDD* major depressive disorder

^a^The prior effect size distribution using the conjugate model with the variance estimated based on the tabulated values of effect sizes reported in Park et al.

^b^The prior effect size distribution specified directly by the estimates reported in Park et al.

## Discussion

It can be argued that regardless of the degree of their variability, *P*-values are poorly suited for what they are used for in practice. Researchers want to know whether a statistic used for summarizing their data supports their scientific hypothesis and to what degree. *P*-values in general do not reflect uncertainty about a hypothesis. This point and other misconceptions have been recently reviewed in a statement on statistical significance and *P*-values by the American Statistical Association^19^.

When using classical intervals, researchers, collectively, may have some assurance that errors would be made at a controlled rate, across the totality of similar studies, but the goal of any individual researcher to quantify statistical support for their hypothesis would be at odds with this long-run coverage property supplied by *P*-intervals. In this regard, *P*-intervals behave statistically in the same way as *P*-values themselves. *P*-values provide long-run error rates control, which is similar to quality control in production. A robot in a production line has a rule for declaring that a part is defective, which allows manufacturers to manage the rate of defective parts that go through undetected. However, the robot is not concerned about whether any *particular* part that goes through the assembly conveyor is defective. In science, on the other hand, an individual researcher has a specific hypothesis at stake. The researcher is naturally more concerned about statistical support for a specific hypothesis of their study than about the average proportion of spurious findings in a journal they are submitting their findings to. I.J. Good made an apt analogy about a statistician that rejects the null hypothesis based on a significant *P*-value that he computed for his client^20^. By doing so, the statistician is protecting his reputation via assurance that after averaging over many clients he will have consulted throughout his career, there will be about *α*% of erroneous rejections of the null hypotheses. On the other hand, the client is at a disadvantage, having no meaningful way of relating that specific *P*-value to the likelihood of being wrong in rejecting the hypothesis. For that, the statistician would have to tell the client the conditional error rate: the fraction of hypotheses that are rejected incorrectly among only those hypotheses that were rejected, but that error rate can only be obtained via a Bayesian approach. The client wants to know whether a statistic used for summarizing the data supports the scientific hypothesis and to what degree, but *P*-values in general do not reflect uncertainty about a hypothesis. In a similar way, endpoints of a specific *P*-interval constructed around a *P*-value obtained in a particular experiment do not generally reflect uncertainty about what a replication *P*-value may be.

Despite their pitfalls, we believe that *P*-values carry useful information that can be supplemented by prior effect size distribution to assess credibility of a summary statistic in a given study. In this article, we focused specifically on variability of *P*-values in replication studies to develop a better appreciation of their potential range, in light of profusion of scientific results that fail to reproduce upon replication. We examined implicit prior assumptions of previously suggested methods and detailed how these assumptions can be explicitly stated in terms of the distribution of the effect size. As an intermediate step of our approach, fully Bayesian posterior distributions for standardized parameters, such as (*μ*_1_−*μ*_2_)/*σ*, are readily extractable from *P*-values that originate from many basic and widely used test statistics, including the normal *Z*-statistics, Student’s *t*-test statistics, chi-square and F-statistics.

Here, we focus on one of many aspects of statistical assessment of replicability; moreover, there are limitations to our approach. While we believe that due to the limited range of possible values for standardized effect size, it is difficult to do worse in terms of mis-specification of the prior distribution than to assume the prior implicit in *P*-intervals, a careful construction of the prior distribution may be difficult—a common issue in Bayesian analysis that is not unique to our particular method. Among other problems are assumptions of the model used to compute *P*-values themselves and including possibilities of confounding unaccounted for by the model. Keeping these limitations in mind, our results show that while classical *P*-intervals are derived without the explicit assumption that all effect sizes are equally likely, such a “flat” prior is assumed implicitly, whenever the endpoints of any given interval are interpreted as related to the range of replication *P*-values, which may lead to bias. For instance, bias will be present if a *P*-interval is constructed for a particular value, such as *P*_obt_ = 0.05. It should be recognized that in some experimental fields, statistical comparisons can be carefully targeted to investigate only those effects that are very likely to be real. A large probability of nearly zero effect size in the prior is inappropriate in this case. Still, one can argue that the prior should reflect some degree of skepticism toward a proposed hypothesis. On the other hand, under the flat prior assumption, all possible effect sizes are equally likely and hence a classical *P*-interval neither contemplates “*a degree of doubt and caution and modesty*”^21^ toward the hypothesis that the effect is present and substantial, nor acknowledges implausibility for the standardized effect size to take large values. When the effect size distribution is modeled in such a way that allows a proportionally small chance to encounter a large effect size and assumes that the majority of effect sizes would be close to zero, the Mixture Bayes approach would explicitly incorporate higher chances of what may be deemed “a false positive result” and it would adjust prediction interval bounds accordingly. Similarly, the flat prior assumption will lead to an invalid *P*-interval if it is constructed for a range of *P*-values (e.g., 0.049 < *P*_obt_ < 0.051). The (1 − *α*)% nominal coverage of *P*-intervals can be Bonferroni-adjusted^5^ for *L* tests as (1 − *α*/*L*)%. While that procedure can restore the long-run coverage property, i.e., (1 − *α*/*L*)% empirical binomial probabilities for *P*-intervals presented in Table 2, the endpoints of such intervals would still lack interpretation as probability bounds for a replication *P*-value. Further, we showed that a flat prior effect size distribution may be incompatible with the bounded nature of the standardized effect size distribution and once again may lead to biased *P*-intervals. For example, many observational studies are seeking for associations between health outcomes and environmental exposures and can be viewed conceptually as a comparison of two-sample means, *δ* = *μ*_1_−*μ*_2_. Presence of a true association in such studies implies a certain difference in mean values of exposure between subjects with and without disease. In such examples, the prior variance reflects the prior spread of the mean of a test statistic, which usually can be related to the spread of the standardized mean difference. The prior spread in units of standard deviation cannot be very large, especially in the fields of observational sciences, that are currently at the focus of the replicability crisis. For example, assuming that effect sizes with the OR greater than three are relatively rare (1% occurrence rate), the prior variance for $ln({\mathrm{OR}})/\sqrt {{\mathrm{Var}}(ln({\mathrm{OR}}))}$ is about 0.01 at its largest possible value (Eq. (15)) and would typically be smaller. Moreover, using the commonly used asymptotically normal statistic for OR as an example, we emphasize that the standardized values *δ* can not exceed approximately −0.66 < *δ* < 0.66 for any value of OR.

Bayesian prediction intervals that acknowledge the actual variability in the possible values of the effect size do depend on the sample size and have correct coverage regardless of whether a selection of *P*-values is present. Reanalysis of the intervals reported by Lazzeroni and colleagues^5^ shows that *P*-intervals can be substantially different from Bayesian prediction intervals, even when sample sizes are very large (Table 4). These results also reflect discrepancies obtained with the direct, “as is” usage of the estimated prior distribution in the Mixture Bayes approach and an attempt to approximate this distribution by the conjugate prior with the same variance. Endpoints of the conjugate intervals on the log scale are comparatively shorter and highlight lack of flexibility inherent in the conjugate approximation to the prior: allowance for a large fraction of effect sizes to be close to zero makes the tails of the conjugate distribution too thin. The estimated prior distribution used by the Mixture Bayes approach is more fat-tailed and is also asymmetric due to a high proportion of minor alleles that carry effects of the positive sign.

**Fig. 3:**
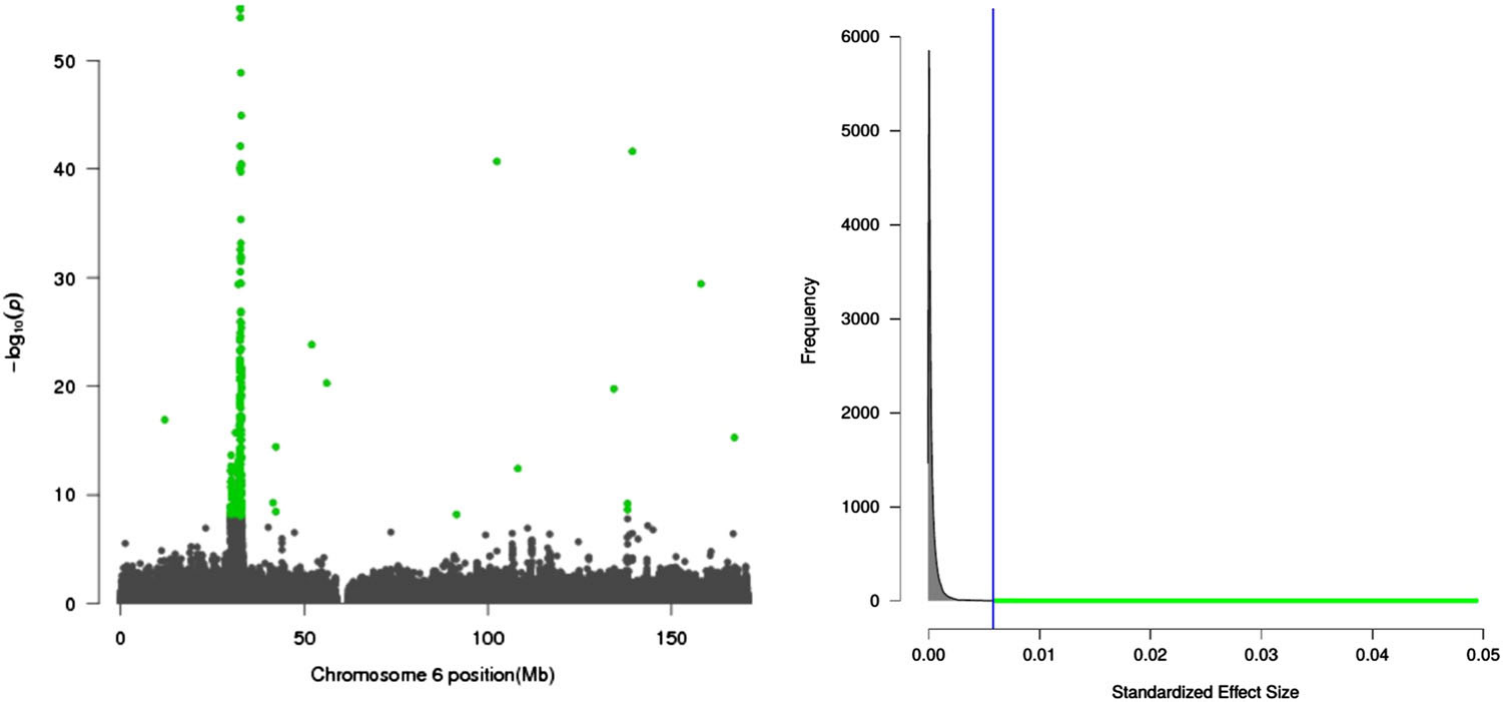
Complex diseases have intrinsically weak genetic effects, as illustrated by a Manhattan plot with only a few significant *P*-values highlighted in green color. The effect sizes corresponding to *P*-values in the Manhattan plot look “L-shaped,” reflecting the idea that the majority of signals are just noise with very little effect sizes (e.g., as measured by $\mathop {{log}}\nolimits^2 ({\mathrm{OR}})$ so the bulk of the effect size distribution is around zero and it is increasingly less likely to find a signal with a large effect size

Bayesian prediction intervals require informed input about various values of the effect size and their respective frequencies. This is not impossible. We know, for example, that in genetic association studies the majority of genetic effects across the genome are tiny and only few are large. This idea can be illustrated by a Manhattan plot in Fig. 3, where the majority of *P*-values are below the significance threshold and only a few hits (highlighted in green color) are deemed to be statistically significant (details about how this Manhattan plot was constructed can be found in the Supplemental section S4). The distribution of the squared effect size (i.e., $\mathop {{log}}\nolimits^2 ({\mathrm{OR}})$ corresponding to those *P*-values is going to be L-shaped^22,23^, as illustrated by the density plot in Fig. 3. We should contrast such an L-shaped prior distribution, even if specified only approximately, with a largely unrealistic assumption implicit in *P*-intervals. When the assumed prior distribution does follow reality, Bayesian prediction intervals enjoy the property of being resistant to selection bias. One can select *P*-values in any range and obtain unbiased intervals or select the minimum *P*-value from an experiment with, however, many tests: the resulting interval would still be unbiased without the need of a multiple-testing adjustment to its coverage level.

It is notable that a major part of *P*-value critique has been revolving around their usage in testing the null hypothesis of the precisely zero effect size, such as *μ* = 0. On the other hand, *P*-intervals of Cumming and Lazzeroni et al. are designed and applied primarily to signed *Z*-statistics for testing one-sided hypotheses, such as *μ* < 0. Indeed, in the context of replication studies, one-sided hypotheses are appropriate, consistent with the goal of replicating the effect direction found in an original study. In fact, one-sided *P*-values can often be related to Bayesian probabilities of hypothesis. Casella and Berger give asymptotic results and bounds for certain statistics^24^. It is also possible to give direct relations in some cases, for example, when testing the mean or mean difference with a *Z*-statistic, the main statistic considered by Cumming and by Lazzeroni and colleagues, and assuming that *a priori*, the mean follows a normal distribution (Suppl. Section S2, Equations S2, S3). One-sided *P*-value for the mean difference between two samples of sizes *n*_1_ and *n*_2_, respectively, is *P*−value = 1 − *F*(*Z*), where *F*(*Z*) is the tail area of the normal curve from—∞ to *Z*. The probability of the null hypothesis given the *P*-value takes a very similar form, $Pr(H_0|{\mathrm{P}} - {\mathrm{value}}) = 1 - F(Z/\sqrt {1 + 1/\left[ {N\vartheta ^2} \right]} )$, where *N* is the half of the harmonic mean of the sample sizes *n*_1_, *n*_2_, and *ϑ* is the variance of a zero-centered prior distribution for the standardized mean. Clearly, the one-sided *P*-value approaches this posterior probability as *N* increases.

Overall, we share the viewpoint of Lazzeroni et al. that *P*-values, or some modifications of them can be useful. Rather than adopting the view that *P*-values should be abandoned because they are poorly suited for what they are used for in practice, we advocate development of statistical methods for extracting information from them in such a way that when augmented with the external (prior) information about the effect size distribution, *P*-value can be transformed into a complete posterior distribution for a standardized effect size. How small a particular *P*-value is (its magnitude) does not inform us what to expect in a replication study^25^. Nevertheless, *P*-values, as transformations of statistics (such as the *Z*-statistic) contain summary information about the standardized effect size. Conditional on that information, one can predict a possible spread of future *P*-values and the respective statistics in replication studies. As part of addressing the multifaceted replicability crisis, researchers would benefit from availability of tools for prediction of variability inherent in commonly used statistics and *P*-values. In particular, prediction intervals equip researchers with quantitative assessment of what they may expect if they would have repeated their statistical analysis using an independent confirmatory sample.

## Electronic supplementary material

Supplementary Material
