# Supplementary material for: Bayesian prediction intervals for assessing P-value variability in prospective replication studies
Source: Transl Psychiatry. 2017 Dec 8;7:1271. doi: 10.1038/s41398-017-0024-3 (PMC5802740; doi:10.1038/s41398-017-0024-3)
Supplement: Supplementary file 1 — Supplementary Material [file 41398_2017_24_MOESM1_ESM.pdf]

Supplementary Information

P-values as random variables

Halsey and colleagues[1] present illustrative simulations for a two-sample t-test, by obtaining random P-values that result from pairs of samples of different sizes N_1, N_2, assuming that the true standardized mean difference between two populations is 0.5. Their graphs and histograms (Figures 3,4 in Halsey et al) underscore substantial randomness in the P-values over repeated samples from the same populations. Indeed, P-values can be viewed as random variables with their respective distributions. For example, P-values derived from common continuous test statistics (such as Student's t) will have the cumulative distribution function (CDF)[2,3] given by $F_\gamma(p) = 1 - G_\gamma\left(G_0^{-1}(1-p)\right)$, where $G_0(\cdot)$ and $G_\gamma(\cdot)$ denote the CDF of the test statistic under the null and the alternative hypotheses and γ is the noncentrality parameter, which in Halsey's et al. experiments is $\gamma = 0.5\sqrt{1/(1/N_1 + 1/N_2)}$. For example, $F_{\gamma=0.5\sqrt{1/(1/30+1/30)}}(0.05/2) = 0.48$, as in Halsey et al.

The probability density function (PDF) of a P-value for a fixed γ follows from differentiating $F_\gamma(\cdot)$, and gives $f_\gamma(p) = \frac{g_\gamma\left(G_0^{-1}(1-p)\right)}{g_0\left(G_0^{-1}(1-p)\right)}$. where $g_\gamma(\cdot)$ is the density that corresponds to the cumulative distribution $G_\gamma(\cdot)$.

The CDF inverse allows to sample random P-values as $P = 1 - G_0\left(G_\gamma^{-1}(U)\right)$, where U is a uniform (0-1) random number. Thus, empirical histograms shown in Halsey's et al. can be reproduced by generating P-values directly, *without simulating the actual samples and computing the t-statistics*. CDF values, $F_\gamma(p)$, would give the expected proportion of P-values in a histogram that are smaller or equal to 'p.' The plot of the PDF, $f_\gamma(p)$, on top of a histogram would match its shape when the histogram is obtained using a large number of simulations. Furthermore, P-value variability can be assessed visually by simply plotting its density. A simple R code implementing this method is available at https://github.com/dmitri-zaykin/Random_P-values.

One-sided P-value of the Z-statistic as a posterior probability of the null hypothesis

P-values for the point null (or short interval) hypotheses, such as $H_0 : 0 < \mu < \delta$ do not correspond to posterior probabilities, but the situation is different in the one-sided testing. Casella and Berger[4] derived general bounds for posterior probabilities for the problem of testing $H_0 : \mu \leq 0$ vs $H_A : \mu >$

0. It is worth to point out that for the conjugate normal model, there is a simple and illustrative connection between P-values for testing this H_0 and the posterior probabilities $\Pr(H_0|Z)$ where Z is the test statistic. Suppose we are testing the difference μ between two sample means, $\mu = m_1$-m_2 and that *a priori*, the mean difference, μ, has a normal distribution, $\mu \sim \Phi(\mu_0, s_0^2)$. The difference $\mu = m_1$-m_2 is the same as (m_1-m_2)-(m_2-m_2). So, we can re-scale to $m_1 \sim \Phi(\mu, s_0^2/2)$, $m_2 \sim \Phi(0, s_0^2/2)$. Given two sample means, $\bar{X}_1$, $\bar{X}_2$, assume normal samples of size N_1, N_2 and the variance σ^2, so that $X_{1,i} \sim \Phi(m_1, \sigma^2)$ and $X_{2,i} \sim \Phi(m_2, \sigma^2)$. We compute the usual Z statistic, $Z = \frac{\sqrt{N}}{\sigma}\left(\bar{X}_1 - \bar{X}_2\right)$, where $N = 1/(1/N_1{+}1/N_2)$. The noncentrality parameter γ for the Z-statistic is a location parameter, $Z \sim \Phi(\gamma, 1)$, and the posterior probability can be derived in terms of the normal density, $f(x \mid \text{mean}, \text{variance})$, as follows:

$$\begin{aligned}\Pr(H_0 \mid Z) &= \frac{\int_{-\infty}^{0} f(t \mid \mu, \sigma^2) f\left[\mu \mid \mu^*, (\sigma^*)^2\right] d\mu}{\int_{-\infty}^{\infty} f(t \mid \mu, \sigma^2) f\left[\mu \mid \mu^*, (\sigma^*)^2\right] d\mu} \\ &= \frac{1}{2}\left[\frac{2}{\sqrt{\pi}} \int_{\omega}^{\infty} \exp\left(-t^2\right) dt\right],\end{aligned}$$

where $\omega = \frac{(\mu^*)\,\sigma^2 + (\sigma^*)^2\, Z}{\sqrt{2}\,\sigma(\sigma^*)\sqrt{\sigma^2 + (\sigma^*)^2}}$; $\mu^* = \mu_0\sqrt{N}$; $\sigma^* = s_0\sqrt{N}$. Considering ω as a location-scale transformation of Z, we can write the posterior probability in terms of the standard normal CDF, $F(\cdot)$ as follows:

$$\Pr(H_0 \mid \text{P-value}) = \Pr(H_0 \mid Z) = 1 - F\left(\frac{Z - \mu_Z}{\sigma_Z}\right), \qquad \text{(S1)}$$

where

$$\begin{aligned}\sigma_Z &= \sqrt{1 + \frac{\sigma^2}{[\sigma^*]^2}} = \sqrt{1 + \frac{\sigma^2}{N s_0^2}} \\ \mu_Z &= -\frac{\mu^* \sigma}{\left[\sqrt{N} s_0\right]^2} = -\frac{\mu_0\,\sigma}{s_0^2\sqrt{N}}.\end{aligned}$$

An impartial prior that gives equal weight to the positive and the negative parts of the distributions, implies that the prior mean for the mean difference is zero, $\mu_0 = 0$. With such a prior, the posterior

probability simplifies to

$$\begin{aligned}\Pr(H_0 \mid \text{P-value}) &= 1 - F\left(\frac{Z}{\sqrt{1+\frac{\sigma^2}{Ns_0^2}}}\right) \\ &= 1 - F\left(\frac{Z}{\sqrt{1+\frac{1}{N\vartheta^2}}}\right) \end{aligned} \tag{S2}$$

where $\vartheta^2 = \left[\frac{s_0}{\sigma}\right]^2$ is the prior variance for the distribution of the standardized mean. As N increases, or as s_0 increases and μ_0 approaches zero, the posterior probability approaches the one-sided P-value, given by

$$\text{P-value} = 1 - F(Z). \tag{S3}$$

Simulations

A statement where P_{obt} is given any value, such as 0.05, is a type of selection that induces selection bias, commonly described as the winner's curse. Our simulations are designed to demonstrate that sort of bias for P-intervals. On the other hand, when we are equipped with knowledge about the actual underlying effect size distribution, the resulting intervals are expected to be immune to selection bias. The most straightforward scenario is to restrict computation of the intervals to P-values in a narrow interval around a value, such as 0.05, and see empirically what the actual coverage is, compared to the declared 80%. We proceeded with this scenario as follows. Let the prior distribution for the mean of a Z-statistic be $\mu \sim \sqrt{N}\Phi(m_0, s_0^2)$. In our simulations we set $m_0 = 0$. Simulation steps are as follows:

1. Draw a value of μ from its assumed distribution. Simulate data by taking two samples of normal observations with the population means 0 and μ and compute a Z-statistic, z_{obt} (this step can be simplified by drawing z_{obt} directly from $\Phi(\mu, 1)$). Calculate the P-value, P_{obt}, from z_{obt}. If P_{obt} does not fall within a specified range, e.g., 0.045 to 0.055, discard z_{obt}. Repeat this step until P_{obt} falls within the predefined range.

2. Simulate data as in the previous step or draw z_{rep} directly from $\Phi(\mu, 1)$.

3. Calculate the P-interval using the prediction distribution $\Phi(z_{\text{obt}}, 2)$. Check whether the replication value falls within the interval.

4. Calculate Bayesian prediction intervals and check whether the replication value falls within these interval.

Next, we repeat the above steps 50,000 times. At the end, we calculate the proportion of times when the replication value was within the studied intervals.

We studied two additional types of P-value selection: (i) inclusion of P-values that are smaller than a predefined threshold, that is, we kept only those P-values that are less than some value, e.g. $P_{\text{obt}} \leq 0.05$; (ii) selection of the minimum P-value from a multiple testing experiment with L tests (in this modification we draw L statistics z_{obt} and keep the maximum one that corresponds to the smallest P-value).

Effect size distribution in genetic association studies

The distribution of the absolute value or squared value of the effect size in genetic association studies is often referred to as "L-shaped".[5] A meta-analysis of six ulcerative colitis genome-wide association studies increased the number of ulcerative colitis-associated loci to 47.[6] The authors of the the study provided results for the discovery panel, which consisted of 6,687 cases and 19,718 controls of European descent for at least 1.1 million SNPs with the corresponding discovery P-values. In our Figure 3, we constructed a Manhattan plot based on the ulcerative colitis-associated SNPs on chromosome 6. Further, in Fig. 3 we highlighted P-values that passed the genome-wide significance threshold (i.e., P-value $< 10^{-8}$) with green color. To obtained the standardized effect sizes (as measured by $\log^2(\text{OR})$) corresponding to this Manhattan plot, we back-transformed each P-values using an inverse chi-squared density function with one degree of freedom. Next, we plotted a histogram of the obtained $\log^2(\text{OR})$ values, which has a distinct "L-shaped" appearance. Such shape, commonly found in genome-wide studies can be described by a mixture of two distributions where the major component accounts for majority of effect sizes that are very small, and the second distributional component allows for occasional variants that carry larger effect sizes than the bulk of the mixture distribution.[7]

Supplementary tables

Type of P-value selection	Prior variance (σ_0^2)	Mixture Bayes coverage
$0 \leq$ P-value ≤ 1	0.25	79.7%
(no selection)	0.50	79.8%
	1.00	80.1%
	3.00	80.1%
	5.00	79.9%
	10.00	79.9%
$0.045 \leq$ P-value ≤ 0.055	0.25	79.4%
	0.50	79.7%
	1.00	80.5%
	3.00	80.5%
	5.00	80.7%
	10.00	79.5%
$0 \leq$ P-value ≤ 0.05	0.25	79.9%
	0.50	79.7%
	1.00	80.7%
	3.00	79.7%
	5.00	80.0%
	10.00	79.9%
$0 \leq$ P-value ≤ 0.001	0.25	79.9%
	0.50	79.0%
	1.00	80.2%
	3.00	80.5%
	5.00	79.9%
	10.00	79.6%

Table S1: The empirical coverage probabilities of the 80% mixture-Bayes prediction intervals for a two-sample t-test under selection of P-values.

Number of tests	Prior variance (σ_0^2)	Mixture Bayes coverage
$L = 10$	0.25	79.2%
	0.50	79.9%
	1.00	80.3%
	3.00	79.8%
	5.00	80.1%
	10.00	79.3%
$L = 100$	0.25	80.3%
	0.50	79.9%
	1.00	79.9%
	3.00	80.7%
	5.00	80.1%
	10.00	80.4%
$L = 1000$	0.25	79.1%
	0.50	80.0%
	1.00	80.4%
	3.00	80.1%
	5.00	79.9%
	10.00	79.9%
$L = 10\,000$	0.25	79.6%
	0.50	80.2%
	1.00	79.2%
	3.00	79.8%
	5.00	79.9%
	10.00	79.3%

Table S2: The empirical coverage probabilities of the 80% mixture-Bayes prediction intervals constructed for the most significant results out of L two-sample t-tests.

References

1. Halsey LG, Curran-Everett D, Vowler SL, Drummond GB. The fickle P value generates irreproducible results. Nat Methods. 2015;12(3):179–185.

2. Kuo CL, Vsevolozhskaya OA, Zaykin DV. Assessing the probability that a finding is genuine for large-scale genetic association studies. PLOS ONE. 2015;10(5):e0124107.

3. Zaykin DV, Zhivotovsky LA. Ranks of genuine associations in whole-genome scans. Genetics. 2005;171(2):813–823.

4. Casella G, Berger RL. Reconciling Bayesian and frequentist evidence in the one-sided testing problem. Journal of the American Statistical Association. 1987;82(397):106–111.

5. Wright A, Charlesworth B, Rudan I, Carothers A, Campbell H. A polygenic basis for late-onset disease. TRENDS in Genetics. 2003;19(2):97–106.

6. Anderson CA, Boucher G, Lees CW, Franke A, D'Amato M, Taylor KD, et al. Meta-analysis identifies 29 additional ulcerative colitis risk loci, increasing the number of confirmed associations to 47. Nature genetics. 2011;43(3):246–252.

7. Chatterjee N, Wheeler B, Sampson J, Hartge P, Chanock SJ, Park JH. Projecting the performance of risk prediction based on polygenic analyses of genome-wide association studies. Nature genetics. 2013;45(4):400–405.
